# Scaling up a Mobile Telemedicine Solution in Botswana: Keys to Sustainability

**DOI:** 10.3389/fpubh.2014.00275

**Published:** 2014-12-11

**Authors:** Kagiso Ndlovu, Ryan Littman-Quinn, Elizabeth Park, Zambo Dikai, Carrie L. Kovarik

**Affiliations:** ^1^Botswana-UPenn Partnership, Gaborone, Botswana; ^2^Department of Dermatology, Perelman School of Medicine, University of Pennsylvania, Philadelphia, PA, USA

**Keywords:** mobile telemedicine, store-and-forward telemedicine, mHealth, public-private partnerships, national eHealth strategy, sustainability, low-resource setting

## Abstract

Effective health care delivery is significantly compromised in an environment where resources, both human and technical, are limited. Botswana’s health care system is one of the many in the African continent with few specialized medical doctors, thereby posing a barrier to patients’ access to health care services. In addition, the traditional landline and non-robust Information Technology (IT) network infrastructure characterized by slow bandwidth still dominates the health care system in Botswana. Upgrading of the landline IT infrastructure to meet today’s health care demands is a tedious, long, and expensive process. Despite these challenges, there still lies hope in health care delivery utilizing wireless telecommunication services. Botswana has recently experienced tremendous growth in the mobile telecommunication industry coupled with an increase in the number of individually owned mobile devices. This growth inspired the Botswana-UPenn Partnership (BUP) to collaborate with local partners to explore using mobile devices as tools to improve access to specialized health care delivery. Pilot studies were conducted across four medical specialties, including radiology, oral medicine, dermatology, and cervical cancer screening. Findings from the studies became vital evidence in support of the first scale-up project of a mobile telemedicine solution in Botswana, also known as “Kgonafalo.” Some technical and social challenges were encountered during the initial studies, such as malfunctioning of mobile devices, accidental damage of devices, and cultural misalignment between IT and healthcare providers. These challenges brought about lessons learnt, including a strong need for unwavering senior management support, establishment of solid local public-private partnerships, and efficient project sustainability plans. Sustainability milestones included the development and signing of a Memorandum of Understanding (MOU) between the Botswana government and a private telecommunications partner, the publication and awarding of the government tender to a local IT company, and the development and signing of a Memorandum of Agreement between the Ministry of Health Clinical Services department and the local tender winner. The initial system scale-up is scheduled to occur in 2014 and to ensure the project’s sustainability, the system is aligned with the national eHealth strategy and local ownership of the project remains at the forefront (1).

## Introduction

Investing in a sustainable telemedicine solution that is relevant to the current health needs is an important milestone any nation would strive to achieve. Nonetheless, making such an investment in a resource-limited environment does not come easy as there are many challenges to overcome including, the buy-in of leadership from key stakeholders. Botswana is the first in Africa to invest in a nationwide mobile telemedicine scale-up project through its Ministry of Health (MoH). This project is in partnership with the Botswana-UPenn Partnership (BUP), the Orange Foundation of Botswana and 5AM Consultancy group. Formal discussion among key project stakeholders began after the completion of the pilot projects in 2010, and in 2012 the MoH officially agreed to scale-up the solution, formally known as “Kgonafalo” (2). The Kgonafalo mobile telemedicine system covers four medical specialties being oral health, dermatology, radiology, and cervical cancer screening, all of which involve visual inspection and follow the same workflow. The Kgonafalo project aims to improve access to specialized healthcare service delivery throughout Botswana hence improving patients’ outcomes and also reducing hospital congestion in the few available referral facilities.

## Background and Problem Statement

Access to specialized health care continues to be a challenge across the African continent (3). This is mostly brought about by the limited number of specialized medical doctors in African countries as compared to developed continents. In order to augment the limited access to health care, many African countries have implemented innovative mobile health (mHealth) pilot project solutions (3). Most of these pilot solutions address public health problems, infectious disease epidemics and pandemics, psychosocial problems such as addiction and suicide, and other social problems such as family planning and smoking cessation, rather than access to specialty care. In addition, the majority of African countries have not yet scaled up mHealth pilot solutions to evaluate their nationwide impact, and no mobile telemedicine programs have been nationally implemented (3). Despite the development of innovative mHealth solutions, there has always been another challenge among many African health systems being that of inefficient information communication technology (ICT) infrastructure characterized by slow bandwidth (3). The recent ICT advancements revived hope for telecommunication technology as a tool to improve health care service delivery through innovative solutions such as telemedicine or telehealth. Telemedicine is the use of telecommunications technology for medical diagnostic, monitoring, and therapeutic purposes where distance and/or time separates the patient and health care provider (4). While others use the broader term telehealth to focus on all health interactions using telecommunications services, many refer to telemedicine in specific domains by using the prefix “tele” with the respective specialty such as teleradiology, teledermatology, and so on. In general, telephone only service is not considered to be true telemedicine (4). The high penetration coupled with low cost of mobile technologies places it well as the ideal telemedicine tool for low-resource settings.

The presence of mobile technology has changed many lives in Africa and nearly two-thirds (65%) of households in 23 countries in sub-Saharan Africa had at least one mobile phone in 2013, with median growth of 27% since 2008, and median annual growth of 5% (5). The 2013 report by the International Telecommunication Union (ITU), predicts that there will soon be as many mobile-cellular subscriptions as people inhabiting the planet, with the figure set to nudge past the seven billion mark early in 2014 (5). The report further states that more than half of all mobile subscriptions are now in Asia, which remains the powerhouse of market growth and by the end of 2013 overall mobile penetration rates will have reached 96% globally, 128% in the developed world, and 89% in developing countries (5). Given the fast growth of mobile technologies, security is a major concern particularly when it comes to health information. Network security standards and protocols for mobile telecommunications have advanced over the past years (6). Internet data security protocols such as the secured version of hyper-text transfer protocol (HTTPS), the Secure Socket Layer (SSL) encryption standard, and the Advanced Encryption Standard (AES) are some of the recommended security measures for the transmission of sensitive data over mobile networks (6). The Health Insurance Portability and Accountability Act (HIPAA) continues to offer encryption standards on Patient Health Information (PHI). The HIPAA demands that all HIPAA covered businesses prevent unauthorized access to “Protected Health Information.” PHI includes patients’ names, addresses, and all information pertaining to the patients’ health and payment records (7).

Some uses of telemedicine include facilitation of access to health care in underserved rural communities especially for specialty care, closer monitoring of patients in their homes in urban or rural settings especially frail elderly or those with chronic disease and “outsourcing” of non-direct care services such as radiology and pathology.

Telemedicine can be classified according to the following categories; office or hospital-based telemedicine, which is an interactive substitute for face-to-face encounter utilizing live video conference with a medical doctor (4). The other type is what is commonly known as the store-and-forward telemedicine, which is purely asynchronous, that is, non-real-time encounter (4). The asynchronous type of telemedicine is more suitable for low-resource settings because of the slow bandwidth challenge. The other telemedicine category is the home-based telemedicine which entails monitoring of patient at their homes or in a nursing care facility (4).

In Botswana, the doctor to patient ratio is at 3.1 per 10,000 and this has resulted in an over-burden of the few specialist doctors available (8). Patients travel long distances hence taking longer times before receiving specialized attention resulting in complex cases and sometimes loss of lives. About 84% of the population in Botswana lives within a 5-km radius and about 95% live within an 8-km radius from a health facility (8).

In 2009, the Botswana MoH introduced the Integrated Patient Management System (IPMS) aimed at improving daily processes in health facilities and also leading to the creation of a single patient health record which is accessible across all health facilities. The full benefits of such an IPMS in Botswana have been hindered by the traditional landline, non-robust ICT infrastructure. Botswana is mentioned to have 87% of households with at least one mobile phone in 2013 and with 3% average annual growth since 2008 (5). Also a significant improvement has been recorded on Botswana’s mobile telecommunication networks such as EDGE, GPRS, 2G, 3G, and the most recent 4G connections. The ICT challenges faced by the MoH in Botswana have inspired innovative ways of offering health care service including a telemedicine solution utilizing mobile devices.

## Pilot Projects

Over the past 4 years, BUP has piloted four mobile telemedicine projects in the specialties of women’s health (cervical cancer screening utilizing visual inspection with acetic acid), radiology, oral medicine, and dermatology (1). Mobile telemedicine has been implemented in 15 locations in Botswana, 27 clinicians have been trained, and 696 cases have been successfully managed (see Table 1).

**Table 1:** **Number of locations, clinicians trained and mHealth cases (January 2011 to September 2013)**.

Specialty	Locations	Clinicians trained	Number of cases
Cervical cancer screening	1	2	356
Teledermatology	2	5	126
Oral telemedicine	10	16	160
Teleradiology	2	4	54
Total	15	27	696

### Pilot project workflow

Healthcare workers were provided with smart phones equipped with a built-in camera and data-enabled subscriber identity module (SIM) cards donated by the Orange Foundation of Botswana. The organizational structure of each mobile telemedicine project includes an in-country medical specialist, an international specialist (the only position that is not held by a MoH employe), a national specialty manager, a referral site coordinator, and the referring health care workers. All four mobile telemedicine projects used the same model in which the healthcare worker collects pertinent clinical history and associated images pertaining to a complex patient case utilizing a smartphone with a telemedicine application (1). Before gathering patient information, a consent form is filled with the patient to allow them to indicate whether they would like to participate in the study or not. The collected history and images are then sent via mobile phones directly by the referring health care worker to an in-country remote specialist for consultation. The specialist uses the information to diagnose the illness and recommend an appropriate course of treatment. The in-country specialist also has the option of forwarding the case to an international specialist for further input and collaboration. The national specialty manager is the uniting force for each program, working to train the health care workers on site, as well as coordinating all parties involved in the scaling up and sustainability of their program.

Some of the benefits identified from the pilot studies include avoiding lengthy waiting times for hospital treatment, cost saving in petrol during referrals, reduction in patient’s long distance travels, and quick medical attention hence improving patient outcomes (1). The pilots also indicate that MoH nurses and other health care workers involved in the scale-up project will gain more knowledge as they regularly interact with the telemedicine solution through uploading of cases and viewing specialists’ responses over time.

In addition, a business research study was conducted by five University of Botswana MBA students in collaboration with University of Pennsylvania Wharton MBA students during the pilot. The purpose of the study was to assess the cost-benefit analysis of a mobile telemedicine solution across all the four medical specialties in Botswana and also propose a sustainable business model. After conducting an economic research analysis, the MBA students found out that the mHealth intervention provided cost savings per patient visit over the first year of implementation.

### Pilot project’s mobile devices

The initial pilot studies utilized the Sony Ericsson C905 to take images and collect patient information on proprietary software from a US-based software company, ClickDiagnostics. All patients’ data were sent to a central database server where specialists accessed them prior to making a diagnosis. Refer Table S1 in the Supplementary Material for a summary of the technical specifications for the Sony Ericsson C905 mobile device.

During 2011, the pilot technology transitioned to use the T-Mobile myTouch 3G Slide Android phones, which allowed partners to use the Open Data Kit (ODK) open-source software supported by a local IT group called PING. Refer Table S2 in the Supplementary Material for technical specifications for the T-Mobile myTouch 3G slide mobile device.

### Pilot project’s mobile application

Customized software from ClickDiagnostics was initially utilized to capture patients’ cases during the pilot project phases. The cases, each consisting of at least thirty unique medical data fields and one to multiple photographs, were sent from the phones over a data-enabled mobile network and stored on a secure database over the internet. The ClickDiagnostics software was later replaced by an open-source mobile application, the ODK, which also captured pertinent patients’ information on mobile smartphones. The choice of the ODK was influenced by the fact that it is free software available on the Internet and has been tried and tested by a community of open-source developers, and it is easy to upgrade without losing data when newer versions are installed. The other advantage with ODK was its flexibility when creating and customizing forms for data entry. The ODK forms also captured patients’ data, which were later saved for transfer to an online database on a cloud based infrastructure.

### Pilot projects objectives and evaluations

Each of the pilot projects focused on specific objectives and was evaluated based on the respective objectives as shown below:

- Cervical Cancer Screening: Evaluating the use of mobile phone telemedicine for cervical cancer screening (9).
- Teledermatology: Determining the reliability and validity of mobile teledermatology in HIV positive patients in a resource-limited setting (10).
- Teledermatology: Evaluating patients’ perception of mobile teledermatology (11).
- Oral telemedicine: Evaluating feasibility of using mobile phone images to diagnose oral medicine cases (12).
- Teleradiology: Evaluating mobile phone image concordance with in-person examination of plain film chest x-rays (13).

## Scale-Up Project

The benefits reported by pilot project users were vital to the adoption of Kgonafalo system into the MoH’s long-term strategy (1). The business study’s promising results coupled with observed improved patient outcomes as a result of the pilot projects, contributed to the MoH support and setting aside funds to support Kgonafalo.

The MoH ultimately committed a budget from the Oral Health Clinical Services recurrent budget to support the Scale-Up of Kgonafalo for oral medicine, dermatology, radiology, and cervical cancer screening.

### Tendering process

In 2013, the MoH Clinical Services published the Invitation to Tender (ITT) documents for supporting the project scale-up (14, 15). This was a public invitation for both local and international companies to submit proposals. BUP facilitated a bidders’ meeting to clarify the needs of the system and answer questions by local IT businesses. Six local companies some of which were in collaboration with international firms submitted their tender proposals.

The three most competitive proposals were evaluated using a template developed by the MoH projects unit. A team of three members conducted the tender evaluation process. The Procurement Department advised on financial evaluations of the proposals and the project’s main contact person came from the MoH Clinical Services Department. The successful proposal evaluation stage led to the tender selection and award process toward the most competitive bidder.

The tender was awarded in December 2013 to 5AM Holdings. The process of developing and publishing the ITT, then evaluating the proposals and choosing the winner occurred over the course of 1 year and 5 months.

Talks with the MoH IT management in January 2014 resulted in an agreement to have the Kgonafalo system hosted on servers at the MoH headquarters. This is one way of ensuring continual system support and ownership of the project by the main stakeholder.

### Public-private partnerships

An MOU was signed in 2013, between the Orange Foundation of Botswana and the MoH. The MOU detailed collaboration roles for each partner in support of the project for the next 3 years. The MOU mentions 19 locations across Botswana as initial project scale-up sites. The Orange Foundation of Botswana committed to donating all mobile devices together with customized SIM cards loaded with data bundles for the project. The process of developing and finalizing the contents of the MOU with respective technical and legal units occurred over the course of 1 year and 7 months.

After the signing of the MOU, The Orange Foundation of Botswana through the support of BUP, identified test mobile devices to be evaluated by all Kgonafalo system users. A successful mobile device evaluation and test session was conducted by specialty managers for the project and user feedback was documented and shared with the Orange Foundation of Botswana.

### Scale-up project’s technical details

Based on feedback from the pilot studies, the BUP provided recommendations to Orange Botswana concerning the specifications requirements for mobile devices for each specialty area. The Orange Foundation of Botswana identified the Alcatel One Touch Idol Ultra 6033X smartphone, in consultation with the BUP. Refer Table S3 in the Supplementary Material for a list of technical details for the selected Alcatel smartphone following test session across the four specialist areas (see Table 2 for feedback from the test sessions).

**Table 2:** **Summary of phone users’ feedback across the four medical specialties**.

	Oral health	Dermatology	Radiology	Cervical cancer screening
Phone screen	Phone screen is the right size	Perfect screen size	The screen size is big enough	Screen is just the right size
Picture quality	Picture quality is excellent	Excellent picture quality	Good pictures captured by the device	The pictures are of good quality
Protective cover	Protective cover is needed	Needs protective cover	Device is fragile and needs some covers	The device needs a cover since it is too slippery
Zoom	Perfect zoom range	Zoom range is just what is needed	Zoom range is about the right length	Zoom range is a bit limited, doesn’t go as far as we would want it
Training	More training needed	Some level of training is needed to first time users	More training required on how to capture perfect images	A lot of training is needed on the touch screen features
Battery	It’s a good device with long battery life compared to previous models	The device is well suited for dermatology and its battery lasts longer	A good phone model with high long battery life	Generally it is a good device, we are happy about the device battery life

In addition to donating mobile devices, the Orange Foundation of Botswana also donated customized SIM data bundles to support connectivity of mobile devices with the system central database. Confidentiality of sensitive patient information is an integral component of the Kgonafalo system. None of the patient’s data is compromised or made accessible through the Orange Botswana servers and network. This has been made possible through the adoption of security protocols such as the SSL encryption, AES, and HIPAA encryption standards on both the mobile application and the enterprise system.

The Orange Foundation of Botswana released an updated network coverage map showing their nationwide 3G, EDGE, and GPRS coverage (16). The Network Coverage Map from the Orange Foundation of Botswana alongside the Kgonafalo expansion locations map are included as part of the Supplementary Material Figure S1.

### Sustainability steps

Sustainability is a major goal of the Kgonafalo project. Sustainability here refers to the life-long operation of Kgonafalo mobile telemedicine solution. Close working relations among the project stakeholders contributed to numerous processes, documents, and presentations being developed to ensure that expansion of the project are conducted responsibly and with a focus on local ownership and drive. In order to achieve this goal, the project partners have developed operational and economical milestones as shown below.

#### Operational milestones

- Information technology (development, maintenance and support) supporting the mHealth system is supported by local IT groups and utilizes open-source software.
- Implementation preparation and training conducted by a local IT group.
- Phone user service-level general support provided by Orange Botswana.
- Phone user service-level SIM card and connectivity support provided by Orange customer service.
- MOU and MOA signed between local public and private partners developed as necessary.
- Policies on mHealth phone usage and staff job descriptions (developed by MoH) that contributed to the project.
- Strategic responsibilities incorporated into the job descriptions of high level MoH, BUP, and Orange positions.

#### Financial milestones

- Pilot/feasibility study operational expenses supported by donors, but smaller expenses (e.g., travel and accommodation for trainings and workshops) shared by local partners.
- All operational expenses of system absorbed by local partners, but donors help support costs associated with transitioning systems to local ownership and providing technical advice and assistance when necessary.
- Long-term roles, responsibilities and benefits of all stakeholders established (Donors can support by conducting cost-benefit analysis and business model research).
- Long-term MOUs and/or operational expenses included in recurrent budgets for local public and private partners.

Efforts have been focused on four main components of sustainability: local customization, local ownership, public-private partnerships (PPP), and the IT provider business model. Empowerment of local ownership and drive are consistently encouraged throughout the scale-up project. A significant amount of planning and major decision making is left with specialty managers and site coordinators who have embraced the projects and pursued initiatives to improve them. The specialty managers organize and run training workshops and sensitization programs for local healthcare workers, allowing them to raise awareness of telemedicine and mHealth. Specialty managers and site coordinators developed the idea of telemedicine awareness posters to hang in all the field sites. Refer Figure S2 in the Supplementary Material for both Setswana and English versions of the mobile oral telemedicine awareness posters. In another step toward local ownership, an official mHealth help desk is currently being developed for all mHealth phone users, which will feed into the existing MoH help desk system. One major sustainable component of Kgonafalo is the fact that it is institutionalized into existing government health programs that can receive budget attention.

## Future Direction

Looking ahead, the MoH is interested in expanding the Kgonafalo project to cover more medical specialties and subsequently cover the whole country. The system has already been built to interface with the MoH electronic medical record system, IPMS; however, there could be more opportunities to interface with more IPMS modules. The data and images captured in Kgonafalo could be used for educational purposes as well as to feed into future decision support systems at the MoH. During the scale up, separate funding will be set aside by the MoH for maintenance and operational support services once the service-level agreement with the local IT group comes to an end.

## Conclusion

BUP, MoH, and the Orange Foundation of Botswana have successfully embarked on a country-wide mobile telemedicine project following successful pilot studies. When not properly planned for and mitigated, scaling up such a project can lead to major setbacks. Kgonafalo has received substantial support from all local stakeholders. The project has attracted local ownership through the PPP engagement. Since the tender award stage, project planning and management by the local partners has been efficient and timelines have been met. The success of the pilot projects and recent scale-up achievements shows that if properly managed, the Kgonafalo solution could be of significant value to Botswana’s healthcare system. The project’s major benefits include substantial reduction of unnecessary referral costs and improved patient outcomes. A number of challenges have been encountered but did not divert stakeholders’ focus from the project’s goals. Recent project developments include the procurement of 42 mobile devices by the Orange Foundation of Botswana and the development of beta version of the mobile telemedicine solution by the engaged local IT group. The success of the scale-up project is largely influenced by senior managements’ support and commitment to the project. Ongoing strong local partnerships and support by specialty managers are equally important for the scale-up project to be a success.

## Conflict of Interest Statement

The authors declare that the research was conducted in the absence of any commercial or financial relationships that could be construed as a potential conflict of interest.

## Supplementary Material

The Supplementary Material for this article can be found online at http://www.frontiersin.org/Journal/10.3389/fpubh.2014.00275/abstract
