# Supplementary material for: Scaling up a Mobile Telemedicine Solution in Botswana: Keys to Sustainability
Source: Front Public Health. 2014 Dec 11;2:275. doi: 10.3389/fpubh.2014.00275 (PMC4269123; doi:10.3389/fpubh.2014.00275)

SUPPLEMENTARY MATERIAL

Table S1: Summary of Sony Ericsson C905 specifications

GENERAL	DISPLAY	MEMORY	CAMERA	BATTERY
**2G Network:** GSM 850 / 900 / 1800 / 1900<br>**3G Network:** HSDPA 2100,<br>HSDPA 850 / 1900 / 2100 - C905a<br>**SIM:** Mini-SIM<br>**Announced:** 2008, June.<br>**Status:** Discontinued. Released 2008, October.	**Type:** TFT, 256K colors<br>**Size:** 240 x 320 pixels, 2.4 inches (~167 ppi pixel density)<br>**Protection:** Scratch-resistant glass	**Internal:** 160 MB internal memory<br>**Card slot:** Memory Stick Micro (M2), up to 8 GB,	**Pixels:** 8.1 MP, 3264 x 2448 pixels, autofocus, xenon flash Geo-tagging, face and smile detection, smart contrast, image stabilization, smile detection (with update),	Li-Po 930 mAh battery (BST-38)<br>**Standby:** Up to 380 h (2G) / Up to 360 h (3G)<br>**Talk time:** Up to 9 h (2G) / Up to 4 h (3G)

Table S2: Summary of T-Mobile myTouch 3G slide specifications

GENERAL	DISPLAY	MEMORY	CAMERA	BATTERY
**2G Network:** GSM 850 / 900 / 1800 / 1900,<br>**3G Network:** HSDPA 1700 / 2100,<br>**SIM:** Mini-SIM<br>**Announced:** 2010, April,<br>**Status:** Available. Released 2010, June.	**Type:** TFT capacitive touchscreen, 65K colors,<br>**Size:** 320 x 480 pixels, 3.4 inches (~170 ppi pixel density),<br>**Protection:** Corning Gorilla Glass	**Internal:** 512 MB RAM, 512 MB ROM,<br>**Card slot:** microSD, up to 16 GB, 8 GB included	**Pixels:** 5MP, 2592X1944 pixels, Autofocus, LED Flash	Li-Ion 1300 mAh battery<br>**Standby:** Up to 286 h,<br>**Talk time:** Up to 6 h 40 min

Table S3: Summary of Alcatel One Touch Idol 6033X specifications

GENERAL	DISPLAY	MEMORY	CAMERA	BATTERY
**2G Network:** GSM 850 / 900 / 1800 / 1900,<br>**3G Network:** HSDPA 900 / 2100, HSDPA 850 / 1900 / 2100<br>**SIM:** Mini-SIM<br>**Announced**: 2013, January<br>**Status:** Available. Released 2013, April.	**Size:** 4.7" HD 720x1280 pixels<br>**Type:** AMOLED 16M color<br>Oleophobic Coating<br>Conrning Glass<br>Multi-touch<br>Proximity sensor<br>Light sensor	**Internal:** 13.2GB MB internal memory<br>**Card slot:** No	**Pixels:** 8 MP<br>Video Player:720p@30fps<br>Video Capture: 720p@30fps<br>Video Codec:H.263, MPEG4,H.264<br>Digital Zoom<br>LED Flash<br>Face and Smile detection	Battery Lithium 1820mAh<br>**Talk time:** 12h(2G)/7h(3G)<br>**Standby time:** 400h(2G)/400h(3G)

Figure S1: Orange Botswana GPRS/EDGE, 3G Network Coverage Map and Kgonafalo scale-up map

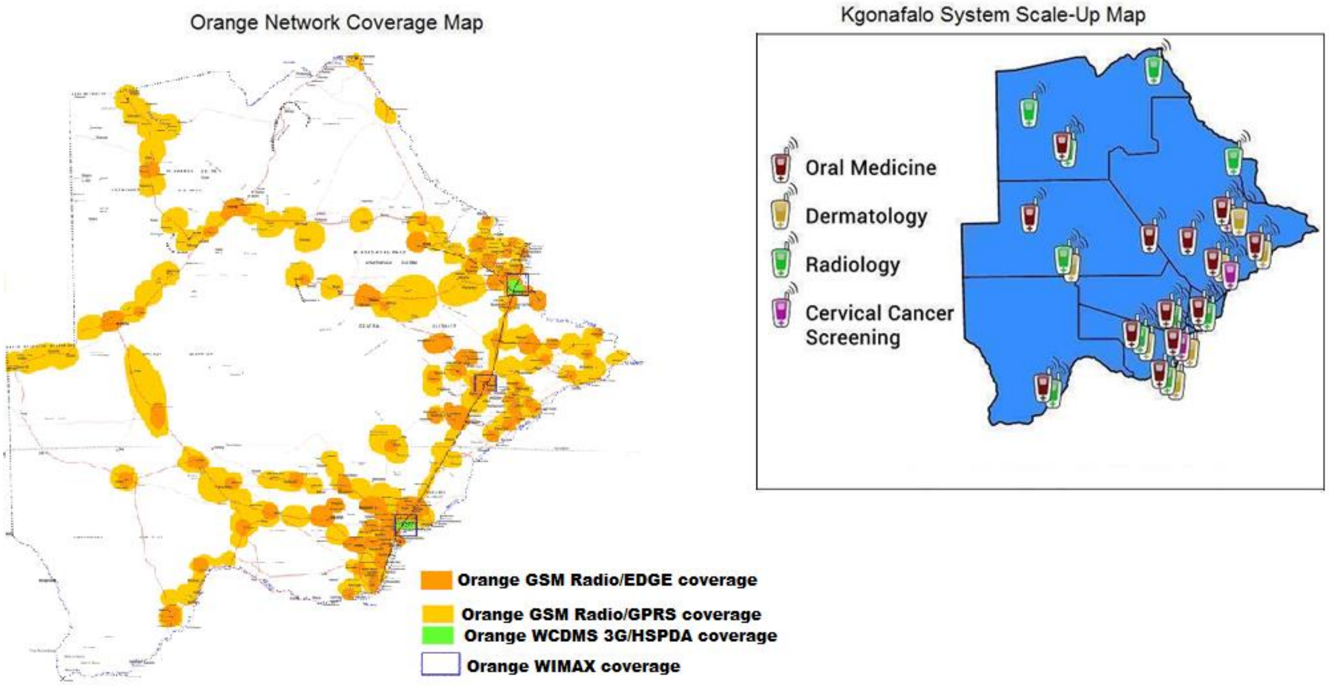

Figure S2: Setswana and English versions of the mobile oral telemedicine awareness posters.

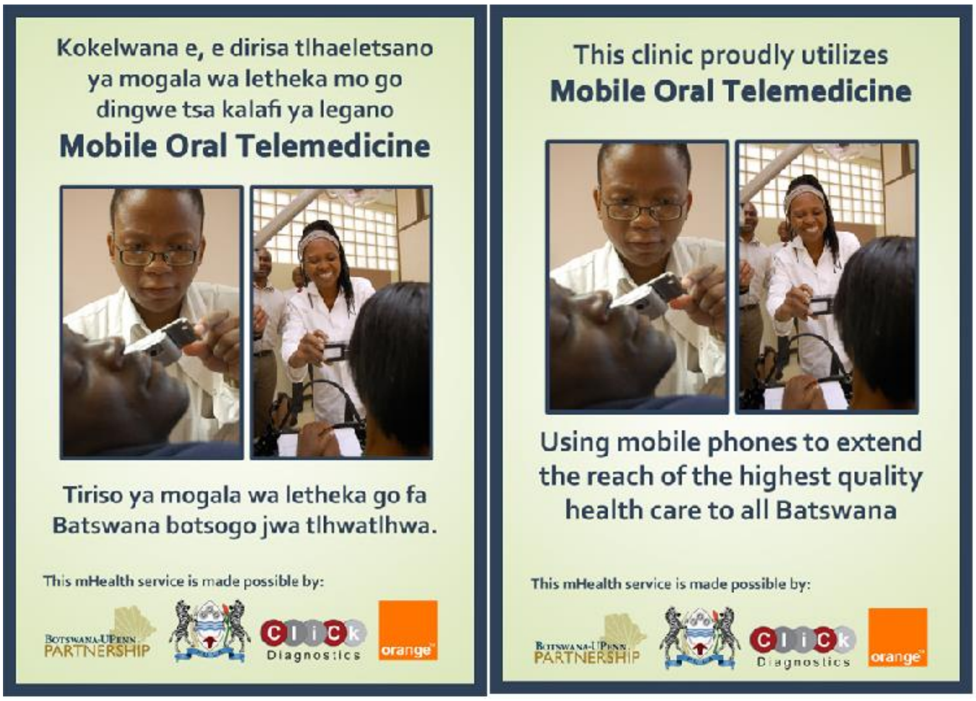

Supplement: Supplementary file 1 [file Presentation_1.PDF]
